# Identification of Genomic Regions Associated with Seedling Frost Tolerance in Sorghum

**DOI:** 10.3390/genes14122117

**Published:** 2023-11-23

**Authors:** Niegel La Borde, Ismail Dweikat

**Affiliations:** Department of Agronomy and Horticulture, University of Nebraska, Lincoln, NE 68583-0915, USA; nlaborde@sacsny.org

**Keywords:** proline, QTL, sweet sorghum, stress tolerance, *Sorghum bicolor*

## Abstract

*Sorghum bicolor* (L.) Moench is the fifth most valuable cereal crop globally. Although sorghum is tolerant to drought and elevated temperatures, it is susceptible to chilling, frost, and freezing stresses. Sorghum seeds planted in April may encounter frequent frost during late April and early May. Early spring freezing temperatures adversely affect crop development and yield. This study aims to identify genomic regions associated with frost tolerance at the seedlings stage. Breeding freeze-tolerant cultivars require selection for freeze tolerance in nurseries. However, the unpredictability of environmental conditions complicates the identification of freeze-tolerant genotypes. An indoor selection protocol has been developed to investigate the genetic determinism of freeze tolerance at the seedling stages and its correlation with several developmental traits. To accomplish this, we used two populations of recombinant inbred lines (RIL) developed from crosses between cold-tolerant (CT19, ICSV700) and cold-sensitive (TX430, M81E) parents. The derived RIL populations were evaluated for single nucleotide polymorphism (SNP) using genotype-by-sequencing (GBS) under controlled environments for their response to freezing stress. Linkage maps were constructed with 464 and 875 SNPs for the CT19 X TX430 (C_1_) and ICSV700 X M81E(C_2_) populations. Using quantitative trait loci (QTL) mapping, we identified six QTLs conferring tolerance to freezing temperatures. One QTL in the C_1_ population and four QTLs in the C_2_ population, explain 17.75–98% of the phenotypic variance of traits measured. Proline leaf content was increased in response to exposing the seedlings to low temperatures. Candidate QTLs identified in this study could be further exploited to develop frost-tolerant cultivars as proxies in marker-assisted breeding, genomic selection, and genetic engineering.

## 1. Introduction

Low temperatures are very physiologically and economically problematic for crops. More than 30% of insurance losses due to weather are due to frost. Global changes in growing conditions due to climate change and an ever-increasing surface temperature lead to an unpredictability of freeze–thaw events during spring and autumn [1,2]. Unexpected frosts are economically devastating in temperate growing areas when they occur in late autumn or early spring. For example, a single frost event in April 2007 destroyed $2.7 billion worth of crops in the Central Plains and Midwest USA [3,4,5]. It has been reported that large-scale freezing events have cost over $31 billion dollars over the last 40 years [6]. More economic losses are caused by frost damage in the USA than by any other weather phenomenon, estimated at $1.5 billion annually [7]. Plants’ response to temperature is critical in determining their environmental distribution and yield. Many of our staple crops originate from climates much different from the zones in which they are currently cultivated. For example, sorghum yields in the US Great Plains are limited to this “thermal threshold of optimal growth” [8]. Sorghum (*Sorghum bicolor* (L.) Moench) is a warm-season cereal, grown in semi-arid, humid, tropical, and sub-tropical areas of the world, with a mean temperature greater than 25 °C during the growing season [9].

Planting sorghum as early as possible in the spring is vital for sorghum production in the US Great Plains. Early planting allows for vegetative growth to happen in cooler temperatures, for reproductive development ahead of the heat stress of the summer months, and for more significant and more profound root growth, which allows for the uptake of subsoil water during the late summer droughts. However, early planting is not without risks; exposure to freezing temperatures, even for short periods, can be catastrophic for sorghum seedlings.

Spring radiation frost occurs under clear night skies when more heat is transferred from the crop than it receives, causing temperatures to fall, which may cause irreversible cellular injury and death, significantly impacting yield and quality [10].

As with most plant species, freezing/frost tolerance in sorghum is a quantitative trait with noticeable variation amongst cultivars. However, the successful exploitation of sorghum’s genetic variation to improve freezing tolerance has been limited. The unreliability of field-based methods for identifying tolerant genotypes is due chiefly to the reduction of observed heritability, owing to environmental variation.

Controlling environmental factors when screening for freezing/frost tolerance may provide a more efficient approach to identifying the genetic variation for frost/freezing tolerance, which can be exploited to produce elite sorghum lines with increased and stable frost/freezing tolerance. Typically, these tests involve subjecting plant tissues, post ice nucleation, to sub-freezing temperatures for specific intervals, thawing at 0–4 °C, and finally evaluating freezing injury based on electrolyte leakage. The freezing stress tolerance is determined as the LT_50_, the temperature at which 50% of plants are injured or die [11]. Temperatures more significant than the LT_50_ are labeled ’sub-lethal’ stress. While simulating natural freezing episodes, these controlled assays often exclude biological variables typical in field conditions [12]. The most prevalent excluded variable is time, as conventionally controlled freezing tests are performed briefly (30–60 min). Notably, it has been shown that prolonged exposure to sub-lethal temperatures is the primary cause of frost-related crop losses [13]. There have been several investigations on the frost tolerance of several grain crops, including wheat, rye, corn, and rice [14]; however, ours is the first indoor frost tolerance study of sorghum.

Proline is the most common endogenous osmolyte accumulated under various abiotic stresses [15]. In response to different pressures, plants accumulate copious quantities of compatible solutes. Compatible solutes are low molecular weight, highly soluble organic compounds that are usually non-toxic at high cellular concentrations. These solutes protect plants from stress by contributing to cellular osmotic adjustment, ROS detoxification, protection of membrane integrity, and enzyme/protein stabilization. These include proline, sucrose, polyols, trehalose, and quaternary ammonium compounds (QACs) such as glycine betaine, alanine betaine, proline betaine, and pipecolate betaine. The accumulation of proline (Pro) is a typical physiological response of many plants to biotic and abiotic stress and is believed to play an adaptive role in plant stress tolerance [16,17]. Recently, evidence has been presented that Pro-stimulated signaling prevents chlorophyll degradation under water stress [18], and there are reports that Pro protects enzymes by modulating electrostatic interactions and stabilizing the tertiary structure [19,20]. Additionally, there is evidence that Pro activates the antioxidative defense directly or through ROS signaling from mitochondrial Pro degradation [21].

The current study aims to identify genetic variation for early spring frost tolerance in sorghum under a controlled environment and to identify chromosomal regions associated with increased frost tolerance. Utilizing two recombinant inbred lines (RIL) populations derived from crossing frost-tolerant sorghum cultivar by frost-susceptible cultivar, we aimed to glean a more robust assessment of freezing response in both grain and sweet sorghum. Identifying QTLs that facilitate freeze tolerance can aid in developing freeze-tolerant sorghum cultivars via maker-assisted breeding.

## 2. Materials and Methods

### 2.1. Genetic Material

The experiment was conducted with two *S. bicolor* F_6_-based RIL populations. A grain sorghum RIL population was created from a cross between CTI9 (chilling-tolerant) and TX430 (chilling-susceptible), coded C_1_. A sweet sorghum RIL population was derived from a cross between ISCV700 (chilling-tolerant) and M81E (chilling-susceptible), coded C_2_. The F_2_ hybrids were developed by hand emasculation of the cold-susceptible line followed by pollination with pollen from the chilling-tolerant lines. The F_2_ hybrids were self-pollinated, and 300 seeds were advanced to the F_7_ stage. A total of 240 C_1_ and 182 C_2_ F6 RIL populations were developed by single seed descent (SSD) in greenhouse conditions. Both mapping populations were bulked at the F_7_ stage at the Lincoln, NE site.

### 2.2. Freezing Tolerance Assay

To examine freezing tolerance, both RIL populations were established in the greenhouse to minimize variations between blocks. Plants were started from seeds in flats of 200 cell “containers” (Steuwe Son, Inc., Corvallis, OR, USA), with one seed per “container.” Each flat was divided into quarters with 50 cells per RIL. Due to space limitations, the experiment was set up as an α-lattice incomplete block design. Each flat was grown under a 16 h (day/night) photoperiod at 30 °C.

### 2.3. Cold Establishment

After the 14-day greenhouse period at 30 °C, which allowed the seeds to emerge and develop to the three-leaf stage, 25 of the seedlings were transferred into the low-temperature incubator for cold acclimation (CA). The acclimation treatment was set at 10 °C for four days followed by 4 °C for three days. Plants were irrigated with chilled distilled water as needed. During the same period, another 25 seedlings were kept in another growth chamber maintained at 25 °C for a non-acclimated treatment (NA). Both cold and non-acclimated treatments were replicated three times.

### 2.4. Growth Analysis

Growth parameters were determined for both CA and NA seedlings before and after three repeated experiments. In each experiment, the fresh weight (FW) and dry weights (DW) of stems were determined. Dry weight was determined after drying fresh stems at 70 °C for 72 h. Shoot length was determined by measuring the distance between a reference mark, above the soil line, and the tip of the leaf before and after CA and NA treatments.

Measurements were performed on 10 plants per NA and CA seedlings. The greenness of the third leaf was determined with a portable SPAD-502 chlorophyll meter (Minolta, Osaka, Japan). The measurements were performed on three parts of the middle section of the third leaf of 10 randomly selected plants per experimental unit.

### 2.5. Evaluation of LT_50_

To determine the LT_50_, electrical changes in the leaf tissue of the frost tolerant and susceptible parental lines for both populations were observed. The freezing tolerance of the NA leaves was determined on the day that seedlings were transferred from the greenhouse (14 days old) to the cold incubator for cold acclimation. Leaves from the cold-acclimated and freezing treatment were evaluated for freezing at the end of the treatment period. Two cm^2^ leaf tissue samples (middle of leaf) were collected from each seedling. The tissue samples were then placed into a test tube containing 100 µL of distilled deionized water. The test tubes were then transferred into a chiller containing glycol for freezing treatment. For non-acclimated tissue, the temperatures were −0.5, −1, −1.5, −2, −2.5, −3, −3.5, and −4 °C. Acclimated tissues were subjected to temperatures of −0.5, −1, −1.5, −2, −2.5, −3, −3.5, −4, −4.5, −5, and −6 °C. Control samples of unfrozen tissue experienced temperatures of −0.5, −1, −1.5, −2, −2.5, −3, −3.5, −4, −4.5, −5, and −6 °C. Control samples of unfrozen tissue were incubated on ice as freezing tolerance experiments were performed. An ice chip was placed into tubes submerged in a glycol bath to initiate ice nucleation at −0.5 °C. Tissue samples in the glycol bath were cooled at a rate of −5 °C every 30 min to final testing temperature (−4 and 6 °C). Tubes were removed from the glycol bath and incubated in ice overnight to thaw, then transferred to 4 °C for 1 hr., and finally incubated for 1 h at room temperature. Twenty (20 mL) of distilled water was added to each test tube then test tubes were shaken at 250 rpm on the platform shaker for 1.5 h. Electrical conductivity (EC) of the leachate was determined with a conductivity meter (Fisher Scientific, Pittsburgh, PA, USA) at room temperature before and after leaf tissues were autoclaved at 121 °C for 20 min. The percentage of ion leakage (IL) was observed as the ratio of initial electrical conductivity (IEC) to final electrical conductivity (FEC). The percent injury was calculated as:

(1) $$\%\;injury_{t}=\frac{\left(\%IL_{t}-\%IL_{C}\right)}{\left(100-\%IL_{C}\right)}\times 100$$

where %IL_t_ and IL_c_ represent the value of % ion leakage at freeze treatment temperature and the non-frozen control. Adjusted injury percentage to maximum injury from freezing: %adjusted injury = (%injury_t_/%injury_max_) × 100 [11]. The percentage injury data from a freezing test were pooled to obtain a mean. LT_50_, the temperature at which 50% injury occurred, was estimated by fitted sigmoidal curves with the Gompertz function.

### 2.6. Freezing Treatment

Ten seedlings per line were transferred from the cold incubator into a chest freezer (Summit Appliance Division, Felix Storch, Inc., Bronx, NY, USA) set at −2 °C. The seedlings were placed in a plastic tray (12*″* × 6*″*× 6*″*) filled with chilled distilled water on top of a container. The seedlings were misted with distilled water to facilitate ice nucleation. The trays were then placed into the chest freezer for 24 h. After 24 h, the trays were removed and placed into a cold incubator (4 °C) for 24 h to thaw. After thawing, the trays were left to recover at room temperature (23 °C) for seven days.

### 2.7. Growth Analysis

Growth parameters for NA and CA seedlings were determined before and after three repeated cold-acclimated trials. During each trial, SPAD, fresh weight, dry weight, and stem length were determined.

### 2.8. Proline Content

Proline content was determined as detailed by Bates et al. [22] and Khed et al. [23]. Bulked leaf tissue from five seedlings was ground via mortar and pestle. Ground leaf tissue (0.5 g) was then homogenized with 10 mL of 3% sulphosalicylic acid and then centrifuged at 10,000× *g*. The resulting supernatant (0.5 mL) was added to a mixture of 1 mL of glacial acetic acid and 1 mL of 2.5% acid ninhydrin (2.5 g ninhydrin dissolved in mixture of 60 mL glacial acetic acid and 40 mL 6 M phosphoric acid), then heated to 100 °C for 1 h. The resulting reaction mixture was cooled on ice for 30 min. The reaction mixture was extracted with 2 mL of toluene and vortexed for 15 s. The chromophore-containing toluene (supernatant) reached room temperature and the absorbance was read at 520 mm using toluene as blank. Proline concentration was determined by standard curve and calculated on a fresh weight basis (µmol proline (gFW)^−1^).

### 2.9. DNA Extraction

The extraction of high-quality DNA from leaf tissue was carried out utilizing a modified protocol proposed by Xin and Chen [24] and previously reported by La Borde, et al. [25].

### 2.10. DNA Quantification and Quality

Genomic DNA (2 µL/sample) was quantified using Thermo Scientific Nanodrop 8000 spectrophotometer instrument (Fisher Scientific). The quality of DNA was then examined by digesting 2 µL genomic DNA (per sample) with HindIII restriction endonuclease Promega, (Madison, WI, USA). DNA pre- and post-digestion was visualized on 1% agarose gel in 1× TBE stained with ethidium bromide, utilizing λ standard DNA dilution series as a control. Samples were stored at −20 °C until shipped to the Institute for Genomic Diversity at Cornell University (now Institute of Biotechnology).

### 2.11. Genotyping-by-Sequencing (GBS)

Genotyping of the C_1_ and C_2_ populations along with their progenitors was performed by the Institute for Genomic Diversity Core facilities according to genotyping-by-sequencing (GBS) protocol described by Elshire et al. [26]. Four 96 well microtiter plates (2 µL per RIL) containing 35 ng of the previously extracted DNA were sent to the Institute for Genomic Diversity where, briefly, the DNA samples were digested using the APeKI restriction enzyme. Then, 96× multiplexed libraries were assembled and sequenced via Illumina Genome Analyzer IIx.

To extract the single nucleotide polymorphisms (SNP) genotypes, the raw reads provided from the Institute were analyzed in the TASSEL software [27] GBS pipeline, and raw reads were aligned to the BTx623 sorghum reference genome. Loci polymorphisms were detected by comparison of consensus sequences from all samples. As RILs were utilized in construction of the libraries, loci with heterozygotes > 10% of total RIL were discarded to reduce false positive results. Only loci with less than 20% missing data were used in mapping.

### 2.12. Linkage Map Construction

Linkage maps for the two populations were created using the SNP data from GBS using R/ASMap [28]. The Kosambi function [29] was utilized to convert recombination fraction into centiMorgans (cM). To detect segregation distortion, chi-square (χ^2^) was calculated using R/ASMap. Unlinked and highly distorted markers were excluded from analysis. The family-wise error rate arising from multiple χ^2^ tests was controlled with the Bonferroni correction. Linkage maps for both C_1_ and C_2_ populations were visualized (with Mapchart 2.3) [30].

### 2.13. QTL Analysis

QTL mapping was carried out to elucidate the genomic regions underlying seedling response to freezing. QTL positions were determined through inclusive composite interval mapping (ICIM) functions of the IciMapping 4.0 software [31]. For all traits, a permutation threshold of 1000 was implemented [32]. The 1-LOD support intervals were set as described by Wang et al. [33]. The results of the QTL analysis for all freeze-related traits, including SPAD and leaf proline content at various temperatures in both C_1_ and C_2_ populations. Additive QTLs having a minimum LOD score of 3.0 were called significant.

### 2.14. Statistical Analysis

The Gompertz function was fitted to the percent injury to determine the LT_50_ of parental sorghum cultivars as described by Lim et al. [11]. The Gompertz equation is defined as:

(2) $$Y=ae^{-be-kx}$$

where a was set at 100, representing maximum injury, **x** represents temperature, e is Euler’s number (2.718…), b fits the x-axis and k fits the y-axis. To evaluate the LT_50_ of sorghum, the experiment was laid out in a complete random design with three replications. The soil-based experiment was laid out in an α-lattice design with two replications for both the C_1_ and the C_2_ population. The analysis of variance was performed with the Agricolae package [34] in the R software [35] using the following equations:

*Y*_ijk_ = *µ* + g_*i*_ + r_j_ + b_ki_ + ε_ij_ (3)

Here, µ is the overall population mean, g_i_ is the genotypic effect, r_j_ is the replication effect, b_k(j)_ is the random block effect nested within the replication effect, and ε_ij_ is the residual effect with a ∼ N (0, σ 2 b). Here, the genotypes were treated as fixed effects, and replication and blocks were considered random effects.

ANOVA was performed on pooled data by treating genotypes as fixed effects and environment (treatment), replication within environments, blocks within environment, and genotype by environment interaction (G × E) effects as random. Pearson’s correlation coefficients among the traits were calculated on a least square mean basis.

## 3. Results

### 3.1. Determination of LT_50_

The parental genotypes varied for percent injury at the non-acclimated (NA) and cold-acclimated (CA) temperatures (Table 1). Non-acclimated leaf tissues suffered a more pronounced injury with decreasing temperatures than acclimated tissues. In addition, the chilling-sensitive parents were observed to suffer more significant injury under decreasing temperatures than the chilling-tolerant parents.

In the C_1_ population, parental cultivar Tx430 expressed freezing injury more than CT19 at all temperature treatments. At −1.5 and −2.0 °C, Tx430 suffered 46.8% and 62.7% greater injury than CT19. The maximum injury for the non-acclimated C_1_ parents was 97.8% for TX430 and 84.9% for CT19 at −5.5 °C. For the C_2_ population, ISCV700 was observed with more significant freezing injury at all treatment temperatures. At −1 °C, ISCV700 suffered 55.9% greater injury than M81E. The observed maximum injury of the non-acclimated C_2_ parents was 94.9% for ISCV700 and 84.7% for M81E at −4.0 °C. In both populations, freezing injury progressively increased in severity with decreasing temperatures, with abrupt increases after −1.5 °C. The percent injury was observed to plateau around −3.5.

**Figure 1:**
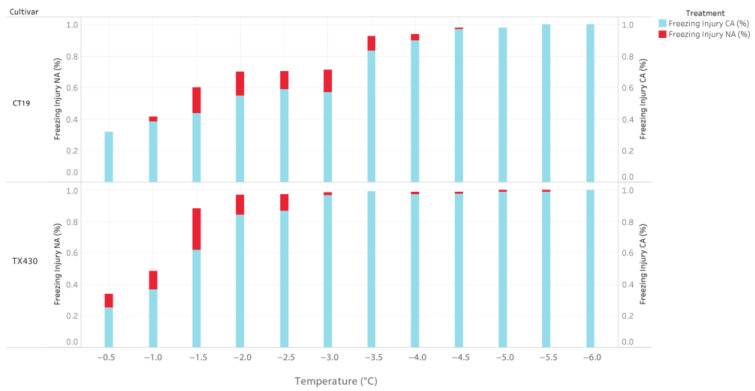
Percentage leaf freezing injury for C_1_ Population, measured at different treatment temperatures (°C). Freezing injury for non-acclimated (NA) and cold-acclimated (CA) by cultivar. Color denotes freezing injury NA (%) and freezing injury CA (%).

**Figure 2:**
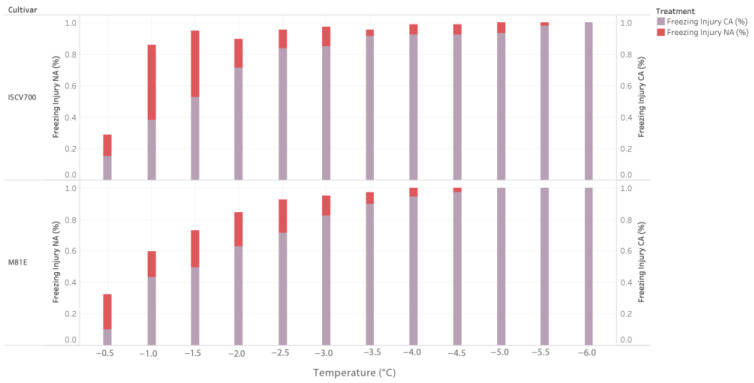
Percentage leaf freezing injury for C_2_ population, measured at different treatment temperatures. Freezing injury for non-acclimated (NA) and cold-acclimated (CA) by cultivar. Color denotes freezing injury NA (%) and freezing injury CA (%).

All parental cultivars demonstrated increased tolerance to freezing temperatures when subjected to a cold acclimation treatment (Figure 1 and Figure 2). In both populations, the chilling-sensitive parent was observed with greater injury to freezing under progressively colder temperatures than the chilling-tolerant parent. In the C_1_ population, CT19 suffered less freezing injury at all temperatures than TX430. At −3 °C, there was a 90% greater freezing injury between CT19 and TX430. The maximum detected injury was 93% for TX430 and 82.1% for CT19 at −5.5 C. In the C_2_ population, the ISCV700 exhibited greater freezing injury at all temperatures than M81E. The maximum observed difference between the cultivars was observed at −1 °C, where a 32.5% difference was observed between ISCV700 and M8IE. LT_50_, the temperature at which 50% of observable tissue injury occurs, was determined using the Gompertz function for both non-acclimated and chilling acclimated parental cultivars. The non-acclimated parents’ LT_50_ values ranged from −0.8I °C (ISCV700) to 1.44 °C (CTI9). Post chilling acclimation, the LT_50_ value decreased for all parents tested, ranging from −1.46 °C (Tx430) to −2.05 °C (CTI9). The frost-tolerant parents had lower LT_50_ values than the cold-sensitive parent at pre- and post-chilling acclimation. For comparing injury under prolonged freezing time (24 h), the mean of the parents’ LT_50_ (−1.6 °C) was selected as the sub-lethal screening temperature.

#### 3.1.1. Effect of Cold Acclimation on Leaf Greenness and Growth

The application of cold acclimation reduced leaf greenness (SPAD) in both the C_1_ and C_2_ populations. On average, subjecting plants to cold acclimation, leaf greenness was decreased by 19.2% in the C_1_ and 26.3% in the C_2_ population. Post freezing treatment, leaf greenness was further reduced in both populations. The C_1_ population was decreased by 27.3% post cold acclimation, representing a 41.3% reduction in leaf greenness. A 26.4% reduction in SPAD was observed post freezing, representing a 46.1% total SPAD reduction in the C_1_ population. There were no significant distinctions in the mean SPAD for plants grown in optimal conditions in both populations. With the application of chilling and frost stress, the chilling-susceptible parent (TX430, ISCV70) in both populations suffered a greater reduction in leaf greenness than the chilling-tolerant parents (CTI9 and M8IE).

The chilling acclimation treatment reduced stem elongation for RILs in the C_1_ and C_2_ populations. In the C_1_ population, mean shoot length for the non-acclimated plants was 20.83 cm while the mean was 12.4 cm post chilling acclimation. The C_2_ population RILs had a mean stem length of 18.11 cm for the non-acclimated seedlings and a mean of 14.I7 cm post chilling acclimation. The C_1_ population was observed with a more severe reduction in stem length than the C_2_ population in response to chilling acclimation.

#### 3.1.2. Changes in Leaf-Free Proline Content in Response to Cold-Acclimation

Leaf proline content in the parental leaf tissue was observed to accumulate progressively under cold acclimation and freezing stress. The chilling-tolerant parents in both populations were kept with greater proline contents at all treatments. In the C_1_ population, the non-acclimatized leaf tissue had a mean proline content of 0.52 µmol proline (gFW)^−1^, while in the C_2_ population, the mean proline content was 0.05I7 µmol proline (gFW)^−1^. The average leaf proline content subsequently increased when seedlings underwent chilling acclimation treatment. In the C_1_ population, this was observed to be 0.560 and 0.115 µmol proline (gFW)^−1^ in the C_2_ population. Under sub-lethal freezing stress, mean leaf proline increased to 0.622 µmol proline (gFW)^−1^ in the C_1_ population and 0.167 µmol proline (gFW)^−1^ in the C_2_ population. The grain sorghum population (C_1_) was observed to have a greater mean leaf proline content than that of the sweet (C_2_) population at all temperatures (Table 2).

**Table 1:** Mean temperature (°C) at which leaf experienced 50% damage (electrolyte leakage). Superscripts represent significant differences amongst cultivars, Tukey’s Test (α = 0.05).

Cultivar	Non-Acclimated (NA)	Chilling-Acclimated (CA)
CT19	−1.44 ^a^	−2.05 ^b^
Tx430	−1.02 ^c^	−1.46 ^ad^
M81E	−1.17 ^de^	−1.75 ^f^
ISCV700	−0.806 ^cg^	−1.53 ^dh^

LT_50_ (°C)

### 3.2. Correlation Analysis

In the C_1_ population (Table 3), significant correlations were found between cold-acclimated tissue leaf greenness (SPAD_CA_) and leaf greenness under freezing stress (SPAD_F_) (0.6076). Additionally, stem length was highly correlated with both cold-acclimated leaf greenness (r = 0.126) and non-acclimated seedlings (r = 0.2359). The free proline content of non-acclimated seedlings was highly correlated with the free proline content of cold-acclimated seedlings (r = 0.6056) and those subjected to freezing stress (r = 0.3311). Similarly, the free proline content of cold-acclimated tissue was highly correlated with seedling proline content post freezing stress (r = 0.631).

In the C_2_ RIL population (Table 4), highly significant correlations were observed between non-acclimated seedling leaf greenness and greenness under both acclimated (r = 0.676) and freezing (r = 0.5112) conditions. Additionally, proline under freezing conditions was significantly correlated with non-acclimated seedling leaf greenness (r = 0.3167). Cold-acclimated seedling leaf greenness was associated with greenness under freezing stress (r = 0.692) and seedling proline content during freezing stress (r = 0.2628). Non-acclimated seedling-free proline content was significantly correlated with cold-acclimated seedling content (r = 0.1943). Cold-acclimated seedling proline content was also correlated with the proline content of seedlings under freezing stress (r = 0.2548).

#### 3.2.1. Linkage Map

The construction of linkage maps for the two populations (C_1_ and C_2_) were assembled from GBS-SNP markers (Figures S1 and S2, Supplementary) with less than 30% missing data. For the C_1_ population, 464 polymorphic GBS-SNPs were mapped to 12 linkage groups. The total map length of 2080.1 cM and covered all ten chromosomes. The individual linkage groups ranged from 57.3 cM to 295.8 cM in length, with an average marker density of 4.6 cM. 875 polymorphic SNP markers were mapped to 14 linkage groups (1515.2 cM total length). All ten chromosomes were covered in linkage groups ranging from 15.03 cM to 242 cM with an average marker density of 1.8 cM per marker.

##### (Frost Damage QTL)

**Figure 3:**
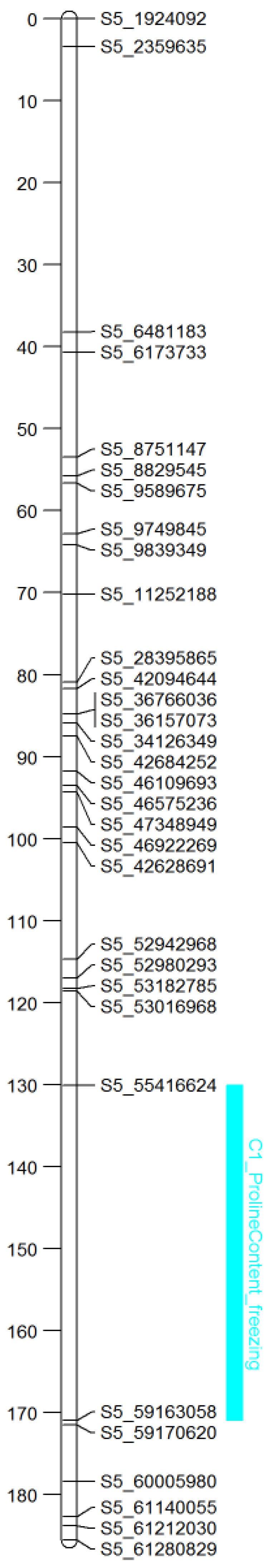
Quantitative trait locus (QTL) mapping of C_1_ population, using 464 linked markers with trait data from chilling and non-chilling-acclimated chilling experiment. Significant traits (LOD > 3.0) considered are: proline content post freezing (leaf-free proline concentration post freezing).

The evaluation for frost-related injuries on the two RIL populations led to the detection of four QTLs in the C_2_ population and 1 QTL in the C_1_ population (Table 5). In the C_1_ population, a QTL for leaf proline content (post-freeze treatment) was discovered on chromosome 5, representing 97.85% of the phenotypic variation in proline content post freezing stress (Figure 3).

**Figure 4:**
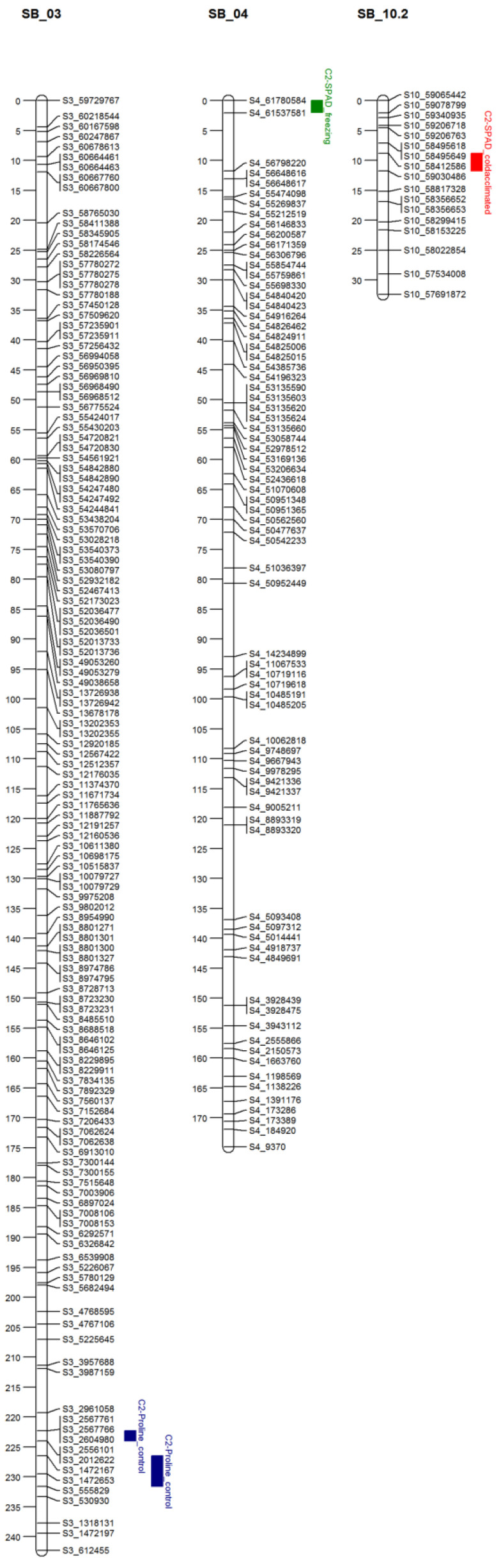
Quantitative trait locus (QTL) mapping of C_2_ population, using 875 linked markers with trait data from chilling and non-chilling-acclimated chilling experiment. Significant traits (LOD > 3.0) considered are proline control (proline concentration in non-acclimated leaves). SPAD freezing (leaf greenness under freezing stress); SPAD cold-acclimated (leaf greenness of cold-acclimated leaf).

In the C_2_ population (Figure 4), one QTL for SPAD_CA_ (post chilling acclimation) was detected on chromosome 10, accounting for 17.75% of phenotypic variation, and led to a reduction in leaf greenness by 1.51. Another QTL for SPAD (post freezing treatment) was observed on chromosome 4, representing 21.93% of the variation in leaf greenness post frost treatment. The allele represented by this QTL led to a reduction of 1.7. Finally, two QTLs for leaf proline content at optimal conditions were identified on chromosome 3. Both QTLs were found within ten cM of one another, one at 233 cM, representing 54.2% of the phenotypic variance, and was associated with a decrease in proline content of (0.002). The other QTL was found at 230 cM, representing 26.1% in phenotypic variance and increased proline content.

## 4. Discussion

Sorghum is a significant crop for food, feed, and biofuels. Developing frost-tolerant cultivars would be crucial for sorghum adaptation in the Great Plains as a fuel crop. Planting sorghum early in the spring promotes higher yields by taking advantage of the early-season moisture and avoiding hotter temperatures during flowering. Low temperatures characterize the growing conditions of sorghum in the Great Plains during the early vegetative stages. Leaf damage via chilling temperatures leading to death is typical. Genetic variation for tolerance to chilling conditions has been observed. Early-season freezing episodes are an abiotic stress that may limit sorghum expansion in the US as a fuel crop. Early-season freezing temperatures not only restrict the sowing time, but seedlings exposed to freezing temperatures suffer adverse effects in development and yield. Significant strides have been made in understanding the genetics underlying Sorghum’s response to chilling temperatures.

Yet, Sorghum’s response to freezing stress has gone virtually ignored, primarily due to the difficulty in evaluating freeze tolerance in the field and the laboratory. Planning for early spring frost episodes at precise growth stages is unpredictable and needs to be reproducible. Simulating natural freeze episodes in the laboratory is problematic, as it is impossible to affect factors such as moisture, wind, ice encasement, radiation cooling, and rhizosphere temperature [12]. A controlled freezing test and a cold acclimation treatment may be a more suitable method, as they minimize said environmental factors [36].

It has been demonstrated that low-temperature stress significantly decreases photosynthetic capacity [37] and rate [38]. It was determined that non-acclimated sorghum seedling leaves accrued freezing tolerance to a maximum of −2.5 °C, while freezing tolerance amongst the parental cultivars varied, and sorghum growth was reduced during the cold acclimation process. This has been previously attributed to a mechanism for stress mitigation [39]. The halting of stem elongation may be attributed to the decrease in the absorption of water and nutrients from the roots during low temperatures [40].

The synthesis and accumulation of proline are thought to be an adaptive response of plants to both biotic and abiotic stresses [41]. We observed increased proline accumulation in leaves under cold acclimation and sub-lethal freezing stress in both populations. Furthermore, increased leaf proline content correlated significantly with leaf chlorophyll content. Previous studies have shown increased proline content, and stress tolerance fluctuated with radiation levels [42].

Sweet sorghum leaves were previously shown to accumulate proline in response to stress, leading to increased osmo-tolerance [43]. This was presumed to be due to the functioning of the SbP5CS gene’s role in the biosynthesis of proline under stress.

### 4.1. Genotyping and Linkage Map Development

Most previously constructed linkage maps for intra-specific Sorghum have utilized SSR markers. However, the identification of polymorphic SSR markers is time- and labor-intensive. As a result, previous studies have been limited to a few hundred tags at best. Next-generation sequencing (NGS) has efficiently constructed high marker density genetic maps concerning time, labor, and costs in recent years. A GBS approach was utilized to identify SNPs and genotyping simultaneously to produce a high-density, low-cost genetic map [44,45]. We identified 1339 novel SNPs between both populations. In comparison to previous GBS studies [26,33,46], in other plant species, we identified fewer SNPs in the present experiment. This may be attributed to the intrinsic disadvantage of GBS-SNP; that is, a large amount of missing data due to narrow sequencing depth.

Additionally, in attempts to capture high-quality data, other SNPs may have been lost by removing SNPs with > 30% missing data. Sequencing errors may have also expanded the genetic distance between markers in the two linkage maps. These extended regions may hinder the discovery of QTLs in such areas [47]. Compared to previous studies, our linkage maps fell into the ranges of those previously published [48,49]. In the C_1_ population, chromosomes, Sb02 and Sb0S and Sb01, Sb05, Sb09, and Sb10 in the C_2_ population were fragmented into two linkage groups. Hiremath [45] reported linkage group fragmentation resulting from high-density genetic mapping and its lack of impact on the fidelity of QTL mapping. From allele analysis, the need for splitting linkage groups was attributed to distorted allele frequencies in the SB02 (C_2_), SB01 (C_2_), and SB10 (C_2_). In addition, large blocks of homogeneity between parental alleles caused other fragments.

### 4.2. Detection of Main Effect QTL

In a large RIL population segregating for freezing tolerance, high power of detection is expected. In addition, co-dominant SNP markers are more informative (via reduction of allelic ambiguity) than dominant markers [50]. Our study utilized two mapping populations, 240 in the C_1_ and 183 in the C_2_ population. 464 (C_1_) and 875 (C_2_) SNP markers were more than requisite for detecting QTLs controlling freezing/frost tolerance and related traits. In writing, this is the first study reporting QTL for freezing/frost tolerance in Sorghum.

A total of one QTL was detected in the C_1_ and four QTLs in the C_2_ population for frost/freezing tolerance-related traits. Among the identified QTLs, three were for proline content and the other two were for leaf chlorophyll (SPAD). All QTLs determined were significant, with a range of genetic variations of 17.78 to 97.8%. This lends credibility when selecting an appropriate screening protocol for these traits.

In the C_2_ population, we found a single major QTL for proline content post-freezing (PRO_F_) stress on chromosome 5, accounting for 98% of the observed variation in seedlings. The interval represented by this QTL includes many genes.

In our study, the QTL detected in the C_1_ population, a significant QTL for leaf greenness under cold acclimation treatment (SPAD_CA_), explaining 17.75 of the phenotypic variances, was seen on chromosome 10. This region was previously identified by Gelli et al. [51] for leaf greenness at vegetative growth, maturity, and grain yield (under normal nitrogen conditions). This region for leaf greenness was also identified by Bekele et al. [52] for leaf greenness for two-week-old seedlings under irrigated and non-irrigated conditions. The occurrence of this region across several populations and environments indicates that this region encompasses genes regulating leaf greenness under both daily and stressful situations. This region contained several genes controlling metabolic processes (Sb10go28770), response to freezing (Sb10go28660), and chloroplast functions (Sb10go28550). Another major QTL for leaf greenness post freezing treatment was found on chromosome 4, accounting for 22% of the phenotypic variance. This region is co-localized with a region identified for the leaf greenness of two-week-old seedlings under irrigated conditions by Bekele et al. [52]. The identified region was found to encompass genes regulating chloroplast (Sb04go02920), chlorophyll and catabolism (Sb04go02990), and the photosynthetic (Sb04go04770) mechanisms of other genes located in this area. On chromosome 3, we identified two significant QTLs for proline content, combining for 80% of the phenotypic variance at control conditions (30). As stated earlier, these QTLs are co-located with one another, indicating that this region encompasses a gene-controlling leaf proline content in Sorghum. Chromosome 3 has previously been shown to contain two genes for ∆ I-pyrroline-5-carboxylase synthetase (P5CS) under osmotic stress [53]. The identified area also had two putative genes (Sb03g002670 and Sb03g002700). Both are uncharacterized to date but are highly expressed in sorghum leaf tissues [54].

## 5. Conclusions

In the current study, we utilized an indoor method to screen Sorghum for freezing tolerance (FT). This method may help hasten the breeding for field frost tolerance in Sorghum. By using an indoor screening method to select sorghum germplasm before planting in the field for frost tolerance, we can reduce the time and resources needed to choose superior germplasm. We have identified six QTLs for FT-related traits in the two populations tested. The most promising was near chromosome 5. Frost-tolerant sorghum cultivars would allow growers to plant earlier without the threat of damage or loss of their crops. Early-season chilling stress and freezing are significant limitations to Sorghum productivity in the Great Plains. Freezing tolerance in Sorghum is a genetically complex trait. However, it may be possible to develop genetically tolerant sorghum germplasm. The results of this study increase the understanding of Sorghum’s physiological responses and genetics to freezing stress and cold acclimation. The ability of sorghum leaves to undergo cold acclimation and tolerate freezing stress was demonstrated by the observable cold-induced changes in leaf greenness, stem growth, and free proline content.

## Figures and Tables

**Table 2:** Indoor Physiological Cold-Acclimation Freezing Response Data for C_1_ and C_2_ Populations.

	Population C_1_				Population C_2_			
	Parents		RILs		Parents		RILs	
Traits	CT19	Tx430	Mean ± SD	Range	M81E	ISCV700	Mean ± SD	Range
SPAD_NA_ *	24.8	17.6	19.57 ± 4.63	8.9–33.6	24.2	17.3	18.55 ± 3.83	8.2–32.1
SPAD_CA_ *	23.6	13.0	14.69 ± 3.6	7.2–27.0	18.5	10.6	13.6 ± 3.6	4.2–24.8
SPAD_F_ *	15.7	9.2	10.54 ± 3.74	1.8–22.5	13.7	8.2	10.0 ± 3.77	1.8–22.5
Pro_NA_ *	0.499	0.4434	0.52 ± 0.0296	0.411–0.599	0.064	0.051	0.0517 ± 0.03	0.004–0.17
Pro_CA_ *	0.5689	0.5403	0.560 ± 0.038	0.481–0.707	0.147	0.08	0.115 ± 0.017	0.15–0.91
Pro_F_ *	0.6531	0.6149	0.622 ± 0.057	0.502–0.817	0.233	0.117	0.164 ± 0.087	0.27–0.52
Stm_NA_ *	17.9	14.14	20.8 ± 4.3	9.62–24.2	23.58	17.3	18.11 ± 4.4	0.00–29.0
Stm_CA_ *	11.7	16.32	12.4 ± 3.5	3.66–20.5	15.16	9.62	14.17 ± 3.34	0.0–22.9

SPAD_NA_—mean chlorophyl content of third leaf for non-acclimated seedling g; SPAD_CA_—mean chlorophyl content of third leaf for chilling acclimated seedlings; SPAD_F_—mean chlorophyl content of third leaf for post freezing seedlings; Pro_NA_—proline content on non-acclimated seedling (µmol proline (gFW)^−1^); Pro_CA_—proline content on chilling acclimated seedling (µmol proline (gFW)^−1^); Pro_F_—proline content on post freezing seedling (µmol proline (gFW)^−1^); STM_NA_—stem length of non-acclimated seedling (cm); STM_CA_—stem length of chilling acclimated seedling (cm). * Indicates significance at *p* ≤ 0.05.

**Table 3:** Pearson’s Correlation of Physiological Traits in C_1_ Population.

	SPAD_NA_	SPAD_CA_	SPAD_F_	Stm_Na_	Pro_Na_	Pro_CA_	Pro_F_
SPAD_NA_	1						
SPAD_CA_	0.1126	1					
SPAD_F_	0.0504	0.6076 ***	1				
Stm_NA_	0.126 *	0.2359 ***	−0.0697	1			
Pro_NA_	−0.0161	−0.0788	0.0365	0.0178	1		
Pro_CA_	0.0626	−0.0477	−0.0795	−0.0619	0.6056 ***	1	
Pro_F_	0.0611	−0.0678	−0.1117	−0.0351	0.3311 ***	0.6731 ***	1

Correlation is significant at: * = *p* ≤ 0.05; *** = *p* ≤ 0.001.

**Table 4:** Pearson’s Physiological Correlation of Traits in C_2_ Population.

	SPAD_NA_	SPAD_CA_	SPAD_F_	Stm_NA_	Stm_CA_	Pro_NA_	Pro_CA_	Pro_F_
SPAD_NA_	1							
SPAD_CA_	0.676 ***	1						
SPAD_F_	0.0511 ***	0.692 ***	1					
Stm_NA_	0.0078	0.0646	0.1978	1				
Stm_CA_	0.0095	−0.104	−0.0747	0.1216	1			
Pro_NA_	0.0499	0.0247	−0.0087	−0.0643	0.0059	1		
Pro_CA_	0.0717	0.1095	0.0074	−0.0092	−0.0565	0.0194 *	1	
Pro_F_	0.3167 **	0.2628 **	0.1396	−0.0847	0.0789	0.1715	0.2548 **	1

Correlation is significant at: * = *p* ≤ 0.05; ** = *p* ≤ 0.01; *** = *p* ≤ 0.001.

**Table 5:** Summary of main effect QTL positions in the C_1_ (n = 240) and C_2_ (n = 183) populations from freezing stress tolerance. QTL (PROF—leaf proline concentration post freezing, PRO_NA_—leaf proline concentration without cold acclimation, SPAD_CA_—leaf greenness after chilling acclimation, SPAD_F_—leaf greenness after freezing), Linkage Group (LG), QTL positions (Peak), 1-LOD flanking markers, additive effects (AE), and percent of explained phenotypic variance (PVE).

Population	QTL	LG	Peak cM	Marker Interval	AE	LOD	PVE
C_1_	PROF	SB_05	145	S5_55416624/S5_59163058	−0.48	33.85	98.12
C_2_	SPAD_CA_	SB_10.2	11	S10_58412586/S10_59030486	−1.51	3.86	17.75
	SPAD_F_	SB_04	5	S4_61780584/S4_61537581	−1.68	4.68	21.93
	PRO_NA_	SB_03	223	S3_2604980/S3_2556101	−0.02	9.22	54.21
	PRO_NA_	SB_03	230	S3_1472653/S3_555829	0.01	5.07	26.13

## Data Availability

The data presented in this study are openly available in FigShare at https://doi.org/10.6084/m9.figshare.22220416.

## Supplementary Materials

The following supporting information can be downloaded at: https://www.mdpi.com/article/10.3390/genes14122117/s1, Figure S1: Genetic linkage map generated using single nucleotide polymorphism markers in 240 sorghum lines from the recombinant inbred line population CT19 × TX430. A total of 464 markers were mapped into 12 linkage groups, with a total linkage distance of 2080.1 cM; Figure S2: Genetic linkage map generated using single nucleotide polymorphism markers in 183 sorghum lines from the recombinant inbred line population M81E × ISCV700. A total of 875 markers were mapped into 14 linkage groups, with a total linkage distance of 1515.2 cM.
