# Supplementary material for: Identification of Genomic Regions Associated with Seedling Frost Tolerance in Sorghum
Source: Genes (Basel). 2023 Nov 23;14(12):2117. doi: 10.3390/genes14122117 (PMC10743030; doi:10.3390/genes14122117)

Figure S1: Genetic linkage map generated using single nucleotide polymorphism markers in 240 sorghum lines from the recombinant inbred line population CT19 x TX430. A total of 464 markers were mapped into 12 linkage groups, with a total linkage distance of 2080.1cM.

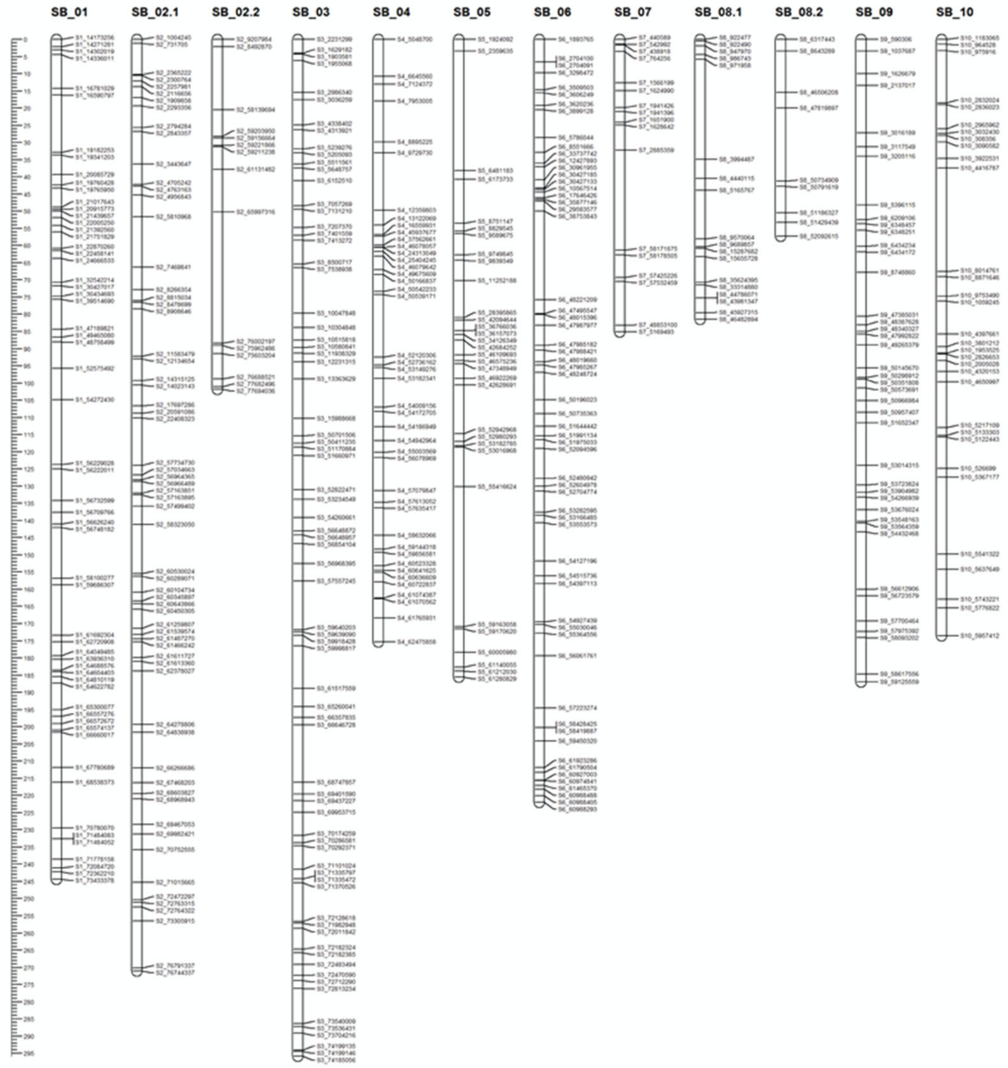

Figure S2: Genetic linkage map generated using single nucleotide polymorphism markers in 183 sorghum lines from the recombinant inbred line population M81E x ISCV700. A total of 875 markers were mapped into 14 linkage groups, with a total linkage distance of 1515.2 cM.

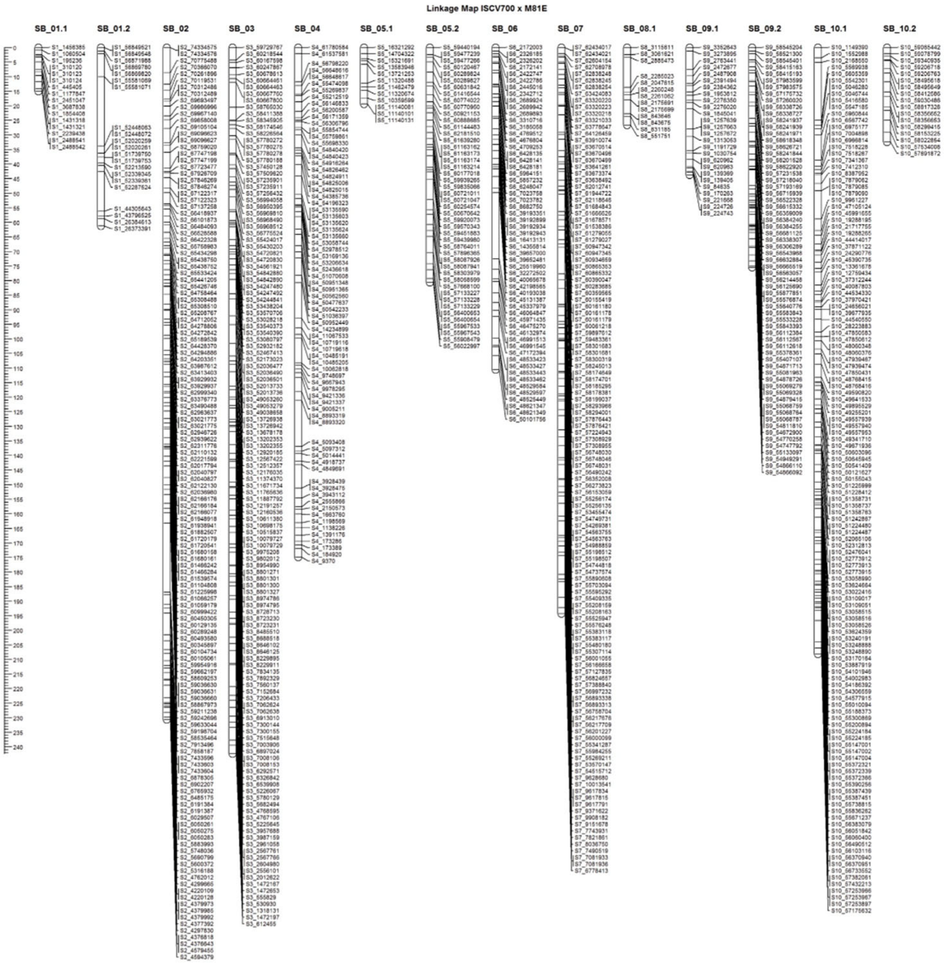

Supplement: Supplementary file 1 [file genes-14-02117-s001.zip › genes-2721188-supplementary.pdf]
